# Tolerance of Transplastomic Tobacco Plants Overexpressing a Theta Class Glutathione Transferase to Abiotic and Oxidative Stresses

**DOI:** 10.3389/fpls.2018.01861

**Published:** 2019-01-11

**Authors:** Evangelia Stavridou, Michail Michailidis, Stella Gedeon, Antri Ioakeim, Stefanos Kostas, Evangelia Chronopoulou, Nikolaos E. Labrou, Robert Edwards, Anil Day, Irini Nianiou-Obeidat, Panagiotis Madesis

**Affiliations:** ^1^Institute of Applied Biosciences, Centre for Research & Technology Hellas, Thessaloniki, Greece; ^2^Laboratory of Pomology, Department of Horticulture, School of Agriculture, Aristotle University of Thessaloniki, Thessaloniki, Greece; ^3^Department of Botany, School of Biology, Aristotle University of Thessaloniki, Thessaloniki, Greece; ^4^Laboratory of Ornamental Plants, School of Agriculture, Forestry and Natural Environment, Aristotle University of Thessaloniki, Thessaloniki, Greece; ^5^Laboratory of Enzyme Technology, Department of Biotechnology, School of Food, Biotechnology and Development, Agricultural University of Athens, Athens, Greece; ^6^School of Natural and Environmental Sciences, Faculty of Science, Agriculture and Engineering, Newcastle University, Newcastle upon Tyne, United Kingdom; ^7^School of Biological Sciences, The University of Manchester, Manchester, United Kingdom; ^8^Laboratory of Genetics and Plant Breeding, School of Agriculture, Forestry and Natural Environment, Aristotle University of Thessaloniki, Thessaloniki, Greece

**Keywords:** chloroplasts, glutathione-S-transferases, tobacco, transplastomics, abiotic stresses, herbicide, transcriptomics, metabolomics

## Abstract

Chloroplasts are organelles subjected to extreme oxidative stress conditions. Biomolecules produced in the chloroplasts act as signals guiding plant metabolism toward stress tolerance and play a major role in regulating gene expression in the nucleus. Herein, we used transplastomic plants as an alternative approach to expression of transgenes in the nucleus for conferring stress tolerance to abiotic stresses and herbicides. To investigate the morphophysiological and molecular mechanisms and the role of plastid expressed GSTs in tobacco stress detoxification and stress tolerance, we used transplastomic tobacco lines overexpressing a theta class *glutathione transferase* (GST) in chloroplasts. The transplastomic plants were tested under drought (0, 100, and 200 mM mannitol) and salinity (0, 150, and 300 mM NaCl) *in vitro*, and under herbicide stress (Diquat). Our results suggest that pt^*At*GSTT^ lines were tolerant to herbicide-induced oxidative and salinity stresses and showed enhanced response tolerance to mannitol-induced osmotic stress compared to WT plants. Overexpression of the *Arabidopsis thaliana At*GSTT in the chloroplasts resulted in enhanced photo-tolerance and turgor maintenance under stress. Whole-genome transcriptome analysis revealed that genes related to stress tolerance, were upregulated in pt^*At*GSTT^2a line under both control and high mannitol stress conditions. Transplastomic plants overexpressing the pt^*At*GSTT^2a in the chloroplast showed a state of acclimation to stress, as only limited number of genes were upregulated in the pt^*At*GSTT^2a transplastomic line compared to WT under stress conditions while at the same time genes related to stress tolerance were upregulated in pt^*At*GSTT^2a plants compared to WT in stress-free conditions. In parallel, the metabolic profile indicated limited perturbations of the metabolic homeostasis in the transplastomic lines and greater accumulation of mannitol, and soluble sugars under high mannitol stress. Therefore, transplastomic lines seem to be in a state of acclimation to stress under stress-free conditions, which was maintained even under high mannitol stress. The results help to elucidate the role of GSTs in plant abiotic stress tolerance and the underlying mechanisms of the GSTs expressed in the chloroplast, toward environmental resilience of cultivated crops.

## Introduction

Developing crop plants, able to yield better under abiotic stresses or plants with multiple herbicide resistance, is a prerequisite for improved crop production. The chloroplast, abundant in plant cells and eukaryotic algae, is the site of photosynthesis, providing the primary source of the world's food productivity (Verma and Daniell, 2007). As chloroplasts are the organelles responsible for photosynthesis they are also a source of reactive oxygen species (ROS) in plants (Foyer and Shigeoka, 2011). Furthermore, environmental stresses have been found to produce an excess of excitation energy in chloroplasts, resulting in the production of ROS, thus they are also considered to be implicated in the regulation of stress responses or even act as a sensor of cellular stress (Mullineaux and Karpinski, 2002). Genetic transformation of chloroplasts has been used as an alternative approach to the expression of transgenes in the nucleus (Wang et al., 2009). The transplastomic system has three main advantages: (i) prevents gene flow via pollen through transgene containment due to maternal inheritance, (ii) has highly active chloroplast transcription and translation machineries, and (iii) a lack of epigenetic interference allows stable transgene expression (Bock, 2014). Chloroplast engineering has been applied for the development of resistant crops to various abiotic and biotic stresses (Clarke and Daniell, 2011), production of biopharmaceuticals, metabolic pathway engineering and advances on RNA editing (reviewed in Wang et al., 2009) and phytoremediation (reviewed in Verma and Daniell, 2007).

Understanding the adaptation of plants to different climatic conditions, such as high temperatures, water logging, and drought is essential for addressing climate change challenges. Improving the resilience of chloroplasts through plastid engineering may provide a solution toward the improvement of crop productivity (Clarke and Daniell, 2011). To date, there are a limited number of studies regarding the development of transplastomic plants and their response to abiotic stress. Transplastomic tobacco plants expressing a choline monooxygenase (*BvCMO*) from *Beta vulgaris* demonstrated increased tolerance to salt (100 and 150 mM NaCl) and drought (300 mM mannitol) stresses (Zhang et al., 2008). Genetic engineering of carrot chloroplast genome expressing the *Betaine-aldehyde dehydrogenase* (*badh)* gene also improved tolerance to high salinity (400 mM L^−1^ NaCl) (Kumar et al., 2004). Similarly, transplastomic *Nicotiana benthamiana* plants expressing multiple defense genes encoding protease inhibitors and chitinase were more tolerant to 200 mM NaCl and 3% PEG compared to the wild type plants and were able to maintain greater root growth activity due to transgene expression in the leucoplasts of roots (Chen et al., 2014). Transplastomic tobacco lines overexpressing an *A. thaliana* γ*-tocopherol methyltransferase* (*At*γ*-tmt)* gene accumulated higher levels of α-tocopherol when grown in 400 mM NaCl, compared to wild-type plants, which accumulated higher starch and total soluble sugars, but transplastomic plants better regulated sugar transport (Jin and Daniell, 2014). Genetically engineered plastomes have provided a generation of herbicide-tolerant plants demonstrated in tobacco for tolerance to glyphosate (Ye et al., 2001; Chin et al., 2003), phosphinothricin (Iamtham and Day, 2000; Lutz et al., 2001) sulcotrione (Falk et al., 2005), isoxaflutole (IFT) (Dufourmantel et al., 2007) and paraquat (methyl-viologen) (Poage et al., 2011; Chen et al., 2014).

Plant glutathione S-transferases (GSTs) have been shown to modulate redox homeostasis by alterations in GSH content and redox state (Sappl et al., 2009), conferring tolerance to a wide range of abiotic stresses (Kumar et al., 2013; Csiszár et al., 2014; Kissoudis et al., 2015b; Kayum et al., 2018) including herbicides (Kissoudis et al., 2015a; Lo Cicero et al., 2015, 2017). Glutathione transferases (GSTs; EC 2.5.1.18) are a superfamily of multifunctional proteins that in plants, have evolved into six discreet groups classified as the zeta (Z), theta (T), phi (F), tau (U), lambda (L), and dehydroascorbate reductase (DHAR) classes, respectively, (Dixon and Edwards, 2010). Functions ascribed to date include the detoxification of herbicides (phi and tau), tyrosine degradation (zeta), the reduction of intermediates involved in redox cycling (DHAR and lambda), and acting as glutathione peroxidases toward organic hydroperoxides (theta). In the case of the theta enzymes (GSTTs), this ability to use glutathione to reduce organic hydroperoxides is conserved between plants and animals and is thought to be important in oxidative metabolism, most notably through the processing of phytotoxic oxidized lipids in the peroxisomes (Dixon et al., 2009; Dixon and Edwards, 2010).

GSTs have been used before in chloroplast transformation; The *Sj*GST26 (EC:2.5.1.18), from *Schistomosoma japonicum* (Smith and Johnson, 1988) and His-tagged derivative of the maltose- binding protein (His_6_-MBP) were expressed in tobacco chloroplasts to be used as affinity tags for the rapid purification of chloroplast-expressed proteins (Ahmad et al., 2012). Transplastomic tobacco lines overexpressing glutathione reductase (GR) alone or combined with GST were more tolerant under 10°C, whereas lines overexpressing dehydroascorbate reductase (DHAR) alone or in combination with GR were more sensitive compared to wild type plants (Grant et al., 2014). When these lines were chilled at 4°C and under relatively high photosynthetically active radiation (PAR), all lines were more sensitive compared to wild type plants, indicating that overexpression of the ROS-scavenging enzymes may be dependent on the interaction of light and cold stress (Grant et al., 2014). Transplastomic seedlings expressing either DHAR or an *Escherichia coli* GST B1-1, which has been shown to exhibit a GSH-dependent peroxidase activity against cumene hydroperoxide (Nishida et al., 1994) and proved to be important for bacterial resistance to hydrogen peroxide- induced oxidative stress (Kanai et al., 2006), or a combination of DHAR:GR and GST:GR in chloroplasts were less sensitive to salt (200 mM NaCl) and cold (4°C) compared to wild type seedlings (Le Martret et al., 2011). However, only the simultaneous expression of DHAR:GR and GST:GR conferred tolerance to methyl viologen (MV) (Le Martret et al., 2011). Transplastomic tobacco lines expressing GR in combination with either DHAR or GST (from *E. coli*) exhibited better tolerance to supplemental UV-B than wild type plants (Czégény et al., 2016). The expression of GSTs in compartments where they are not normally found in, can reveal new insights into their functions. For example, the expression of GSTs in cellular compartments (recombinant bacteria, plant chloroplasts) producing porphyrins has revealed their ability to bind to porphyrinogen intermediates (Dixon et al., 2008). In the case of the *Zm*GSTU1-*Zm*GSTU2, the transplastomics ability to protect plants against herbicides that inhibit porphyrin synthesis in the chloroplast shed light into the functional role of the engineered chimeric enzyme (Dixon et al., 2008).

The GSTs are predominantly not targeted for expression in the chloroplast, however, if they are expressed in this organelle, they could deliver some of their key antioxidant and detoxification functions, such as metabolizing photosystem herbicides, and reducing lipid hydroperoxides generated by ROS formed during photosynthesis. In addition to the efficiency of transplastomic expression, we were also interested in how the protective functions of GSTs could be manifested in an organelle where they are not normally targeted for expression. GSTs are important enzymes of the antioxidant pathway and when expressed in the plastome we hypothesized that the leaf physiology and performance would be enhanced under stress compared to the non-transformed wild type plants. None of the above-mentioned examples were performed with plant derived GSTs from the Theta or Tau classes. Therefore, to investigate whether the overexpression of these GSTs in the chloroplast enhances tolerance to salinity, drought, and herbicide induced oxidative stress we used T1 transplastomic tobacco lines overexpressing a theta class GST from *Arabidopsis thaliana At*GSTT1 *(At5g41210)*, an enzyme normally only expressed in the peroxisomes which is highly active as a glutathione peroxidase toward organic hydroperoxide substrates or a *Zea mays* tau class chimeric *Zm*GSTU1*/*ZmGSTU2 enzyme (EFD6-115A), which has been previously shown to protect the transformed plants from herbicide injury through its ability to detoxify fluorodifen (Dixon et al., 2003) and in subsequent studies it was confirmed that the chimera had the additional ability to bind porphyrinogen intermediates formed during chlorophyll biosynthesis, a trait shared with its *Zm*GSTU parent proteins (Dixon et al., 2008). To assess plant tolerance to abiotic stresses, we investigated the morphophysiological parameters, and the metabolic and transcriptomic reactions involved in the response of transplastomic tobacco lines. Herein, we approach plant stress tolerance from an alternative perspective via chloroplast engineering to (i) mitigate the oxidative stress imposed under various abiotic and anthropogenic stress conditions and (ii) unravel the complex networks of molecular interactions controlling plant acclimation to field conditions.

## Materials and Methods

### Plant Material and Experimental Design

For the experiments we used homoplastomic, transplastomic tobacco lines pt^*At*GSTT^ and pt^EFD6−115A^ overexpressing the *At*GSTT (lines 2a and 6-1) or a *ZmGSTU1-ZmGSTU2* chimera in chloroplasts, respectively (Dixon et al., 2008). The seeds of the pt^*At*GSTT^ and pt^EFD6−115A^ T1 lines were initially grown on MS selection medium supplemented with Streptomycin Sulfate (500 mg L^−1^) and Spectinomycin Dihydrochloride (250 mg L^−1^) (Duchefa Biochemie, The Netherlands), whereas the wild-type (WT) tobacco seeds were placed on plain MS medium. After selection, the plantlets were transferred to MS media for further growth and when they reached four true leaves were tested *in vitro* under drought (0, 100, and 200 mM mannitol; AppliChem-PanReac, Germany) and salinity (0, 150, and 300 mM NaCl; Centralchem, Slovakia) conditions (*n* = 6). The experiments lasted for 35 and 20 days, respectively.

The *in vivo* herbicide experiment was performed in a controlled glasshouse environment with a photoperiod of 14/10 h light/dark. The temperature was between 20 and 27°C, with a mean temperature of 23°C. Plantlets undergone acclimatization for 3 weeks and Diquat a non-selective contact herbicide, was applied as Reglone 20 SL formulation (Syngenta Hellas) at 1 and 2 L of Reglone/ha (200-low dose; Diq_L and 400 -high dose; Diq_H, g ai of diquat per hectare, respectively). Herbicide treatments were performed with a portable field plot sprayer (AZO-SPRAYERS, P.O. Box 350-6710 BJ EDE, The Netherlands) using flat-fan nozzles (Teejet Spray System Co., P.O. Box 7900, Wheaton, IL 60188) and calibrated to deliver 300 L/ha of water at 280 kPa pressure.Diquat (REGLONE® Desiccant, Syngenta Canada Inc), Control plants were sprayed with the same volume of water only (no herbicide). All pots were placed in a randomized complete block design (transgenic lines: *n* = 15 and WT: *n* = 9). The experiment lasted for 2 days after herbicide application.

### Morphophysiological Measurements

Dark-adapted chlorophyll *a* fluorescence measurements were performed on the youngest fully developed leaf on the adaxial leaf surface using the OS30p+ chlorophyll fluorometer (Opti-Sciences Inc., Hudson, USA) following dark adaptation of 30 min. Relative chlorophyll content was measured according to Stavridou et al. (2016) on one leaf per plant with three averaged measurements using a CCM-200 plus chlorophyll content meter (Opti-Sciences Inc., Hudson, USA). Harvested plants were separated into leaves, stems, and roots and the final morphological parameters, such as stem length, root length, number of leaves, and plant fresh matter (M_F_) were measured. The plant dry matter (M_D_) was obtained after drying at 60°C until constant weight.

### Transcriptomic Analysis

Total RNA from whole plant tissue of pt^AtGSTT^2a line and WT plants under control and high mannitol stress conditions *in vitro* was isolated using the Monarch Total RNA Miniprep kit (BioLabs Inc., UK) and their concentration was determined spectrophotometrically. The RNA sequencing was performed by the BGI (Denmark). The RNA results were compared as follows: pt^*AtGSTT*^2a and WT in control conditions (groups 1 and 3) and pt^*AtGSTT*^2a and WT in high mannitol (200 mM) stress (groups 2 and 4) (Table 1).

**Table 1:** Plants used for RNA extraction and transcriptomics analysis.

**Analysis group**	**Plant**	**Conditions**
Group 1	pt^*At*GSTT^2a	Control
	pt^*At*GSTT^2a	Control
Group 2	pt^*At*GSTT^2a	Mannitol High
	pt^*At*GSTT^2a	Mannitol High
Group 3	WT	Control
	WT	Control
Group 4	WT	Mannitol High
	WT	Mannitol High

Agilent 2100 Bioanalyzer (Agilent RNA 6000 Nano Kit) was used for the total RNA sample QC: RNA concentration, RIN value, 28S/18S and the fragment length distribution. We use NanoDrop^TM^ to identify the purity of the RNA samples. The first step in the workflow involves purifying the poly-A containing mRNA molecules using poly-T oligo-attached magnetic beads. Following purification, the mRNA is fragmented into small pieces using divalent cations under elevated temperature. The cleaved RNA fragments are copied into first strand cDNA using reverse transcriptase (Takara Bio Inc.) and random primers. This is followed by second strand cDNA synthesis using DNA Polymerase I and RNase H (Takara Bio Inc.). These cDNA fragments then have the addition of a single “A” base and subsequent ligation of the adapter. The products are then purified and enriched with PCR amplification. The PCR yield was quantified by Qubit and the samples were pooled together to make a single strand DNA circle (ssDNA circle), which gave the final library. DNA nanoballs (DNBs) were generated with the ssDNA circle by rolling circle replication (RCR) to enlarge the fluorescent signals at the sequencing process. The DNBs were loaded into the patterned nanoarrays and pair-end reads of 100 bp were read through on the BGISEQ-500 platform for the following data analysis study. For this step, the BGISEQ-500 platform combines the DNA nanoball-based nano arrays and stepwise sequencing using Combinational Probe-Anchor Synthesis Sequencing Method.

### Bioinformatics Workflow

The reads were filtered for low-quality reads (>20% of the bases qualities are lower than 10), reads with adaptors and reads with unknown bases (N bases more than 5%) to get the clean reads using SOAPnuke software. Then we mapped the clean reads onto reference genome, followed by novel gene prediction, SNP & INDEL calling and gene splicing detection. Finally, we identified DEGs (differentially expressed genes) between samples and performed clustering analysis and functional annotations. After filtering, the remaining reads are called “Clean Reads” and stored in FASTQ format.

Regarding Genome Mapping, HISAT (Hierarchical Indexing for Spliced Alignment of Transcripts) was used to do the mapping step (Kim et al., 2015). The StringTie (Pertea et al., 2015) was used to reconstruct transcripts and Cuffcompare [Cufflinks tools- Trapnell et al. (2012)] to compare reconstructed transcripts to reference annotation. After that, the “u,” “I,” “o,” ”j” class code types were used as novel transcripts followed by a support vector machine-based classifier, named Coding Potential Calculator (CPC) (Kong et al., 2007) to predict coding potential of novel transcripts, then the coding novel transcripts were merged with reference transcripts to get a complete reference, and downstream analysis was based on this reference. The clean reads were mapped to reference using Bowtie2 (Langmead and Salzberg, 2012), and then gene expression level was calculated with RSEM (Li and Dewey, 2011), a software package for estimating gene and isoform expression levels from RNA-Seq data. After calculating Pearson's correlation between all samples using cor, hierarchical clustering was performed between all samples using hclust, and PCA analysis with all samples using princomp, and the diagrams were drawn with ggplot2 in R (R Core Team, 2016). The detection of DEGs was performed with NOIseq, which is based on noisy distribution model, as described in Tarazona et al. (2011). The Hierarchical Clustering Analysis of DEGs was performed using heat map function in R. With the GO annotation result, DEGs were classified according to official classification, and GO functional enrichment was also performed using p hyper in R. The *p*-value calculating formula in hypergeometric test is Equation 1:

$$\begin{array}{l} P=1-\overset{m-1}{\underset{i=0}{\sum }}\frac{\left(\begin{array}{c} M\\ i \end{array}\right)\;\left(\begin{array}{c} N-M\\ n-i \end{array}\right)}{\left(\begin{array}{c} N\\ n \end{array}\right)} \end{array}$$

Then the false discovery rate (FDR) for each *p*-value was calculated and in general, the terms which FDR was not larger than 0.01 were defined as significantly enriched. With the KEGG annotation result, we classified DEGs according to official classification, and we also performed pathway functional enrichment using phyper in R with the same *p*-value calculating formula in Equation 1 and the FDR was calculated as described above.

To find the ORF of each DEG the getorf function was used. For plants, ORF were aligned to TF domains (from PlntfDB) using hmmsearch (Mistry et al., 2013). DIAMOND (Buchfink et al., 2014) was used to map the DEGs to the STRING database (von Mering et al., 2005) to obtain the interaction between DEG-encoded proteins using homology with known proteins. The top 100 interaction networks were selected to unfold the pathways involved and for the entire interaction result we provide an input file that can be imported directly into Cytoscape for complex network analysis and visualization.

### Metabolite Extraction, Derivatization, and GC–MS Analysis

Determination of primary polar metabolites was performed as described by Lisec et al. (2006) and Michailidis et al. (2017) with slight modifications. Whole plant lyophilized material (~0.040 gr) from pt^AtGSTT^2a and WT plants under *in vitro* high mannitol stress and control conditions (three biological replicates) were transferred in 2 mL screw cap tubes with 1400 μL of precooled (−20°C) pure methanol. Adonitol (100 μL of 0.2 mg mL^−1^) was added as internal quantitative standard, and incubated for 10 min at 70°C. The supernatant was collected after centrifugation (11000 g, 4°C, 10 min) and 750 μL chloroform (−20°C) plus 1500 μL dH_2_O (4°C) were added. Following centrifugation (2200 g, 4°C, 10 min), 150 μL of the upper polar phase were transferred into a 1.5 mL glass vial and placed under vacuum until drying. Dried residues were re-dissolved by gentle shaking in 40 μL of 20 mg mL^−1^ methoxyamine hydrochloride for 120 min at 37°C, thereafter they were treated with 70 μL of N-methyl-N-(trimethylsilyl) trifluoroacetamide reagent (MSTFA), and incubated for 30 min at 37°C. GC-MS analysis was carried out in Thermo Trace Ultra GC equipped with ISQ MS and TriPlus RSH^TM^ auto-sampler (Thermo Fisher Scientific™, Switzerland). One μL was injected with a split ratio of 70:1. GC separation was held on a TR-5MS capillary column 30 m x 0.25 mm x 0.25 mm (Thermo Fisher Scientific™, Switzerland). Injector temperature was 220°C, ion source 230°C, and the interface 250°C. A constant flow of 1 mL min^−1^ was used for carrier gas. The GC temperature program was held at 70°C for 2 min, then increased to 260°C (rate 8°C min^−1^), where it remained for 18 min. Mass range of m/z 550 was recorded, after 5 min of solvent delay. The mass spectra were acquired in electron impact ionization mode. The peak area integration and chromatogram visualization was performed using the X-calibur processing program. Standards were used for peak identification or NIST11 database (Michailidis et al., 2017) in case of unknown peaks. The detected metabolites were assessed based on the relative response compared to adonitol and expressed as relative abundance.

### Statistical Analysis

All the statistical analyses were performed using the computing environment R. The effects of stress treatments and the genotypes on the morpho-physiological parameters of the *in vitro* experiments and harvesting parameters of the *in vivo* experiments and treatments genotypes and time (days-where applicable) on the physiological parameters were assessed using two-way or three-way ANOVA, respectively, with the *ez* and *afex* packages (Lawrence, 2016; Singmann et al., 2018). All data were tested for normality (Shapiro test) and if normality failed and transformations were attempted. Data were also tested with Mauchly's test for sphericity, and if the assumption of sphericity was violated, the corresponding Greenhouse–Geisser corrections were performed. If significant differences were found among treatments, then the Tukey's HSD *post hoc* test was performed to determine specific treatment differences using the *agricolae* package (de Mendiburu, 2017). For metabolic data, two-way ANOVA was conducted using SPSS (SPSS v21.0., Chicago, USA) and statistically significant differences were based on Duncan's multiple range test (raw data) and Student's *t*-test for comparisons between genotypes or treatments at *P* < 0.05 (Table S1). The raw data are presented in Table S2 and the reported data are relative to the M_F_ of the pt^*At*GSTT^2a line and WT plants.

## Results

### Effect of GST Overexpression on Oxidative Stress Tolerance

Both low and high Diquat concentrations were severe enough to cause chlorotic lesions from day one (Figure S1) and senescence by day 2 on both WT plants and transplastomic lines (Figure S2). Transplastomic lines pt^*At*GSTT^2a and 6.1 showed chlorophyll content with increasing Diquat dose, compared to the WT control, except line pt^EFD6−115A^, which showed reduced chlorophyll content following Diquat exposure (Table 2 and Table S3). Both Diquat doses negatively affected the maximum quantum efficiency of photosystem II (PSII) photochemistry in both WT and transplastomic lines, indicating that the oxidative stress was too severe possibly as a result of extensive free radical formation. The high diquat dose had a more severe effect on the M_F_ of transplastomic line pt^*At*GSTT^ 6.1 (not statistically significant to the control) and WT plants (*p* < 0.05), and a less severe reduction was induced in pt^*At*GSTT^ 2a (not statistically significant to the control) and pt^EFD6−115A^ (*p* < 0.05) (Table 2 and Table S3). M_D_ was not affected by any Diquat dose, indicating that any reduction in M_F_ was a result of turgor loss potentially, inhibiting the respiratory processes due to the function of Diquat as a rapid-acting translocated desiccant (Cronshey, 1961; McNaughton et al., 2015). An increase in M_D_ of pt^*At*GSTT^ 2a was observed under both Diquat doses, however, the M_D_ of WT plants and transplastomic lines pt^*At*GSTT^ 6.1 and pt^EFD6−115A^ was decreased with increasing Diquat concentration (Table 2).

**Table 2:** Percent of change difference in growth (fresh-M_F_ and dry-M_D_ matter; g) and photophysiological parameters (relative chlorophyll content- Chl and Maximum quantum yield of PSII-*F*v/*F*m) of *GST transplastomic* lines and WT tobacco plants grown for 2 days in low (Diq_L) and high (Diq_H) Diquat dose compared to control conditions.

**Genotype**	**Treatment**	**M_**F%**_**	**_**HSD**_**	**M_**D%**_**	**_**HSD**_**	**Chl_**%**_**	**_**HSD**_**	***F*v/*F*m_**%**_**	**_**HSD**_**
pt^*At*GSTT^6.1	Diq_L	−44.86	^a^	−7.41	^a^	24.67	^a^	−41.25	^b^
pt^*At*GSTT^6.1	Diq_H	−51.4	^a^	−23.46	^a^	35.16	^a^	−45	^b^
pt^*At*GSTT^2a	Diq_L	−49.84	^b^	23.73	^a^	−0.28	^a^	−43.04	^b^
pt^*At*GSTT^2a	Diq_H	−30.11	^a^	62.71	^a^	8.52	^a^	−49.37	^b^
pt^EFD6−115A^	Diq_L	−71.95	^b^	−28.23	^a^	−18.87	^ab^	−48.75	^b^
pt^EFD6−115A^	Diq_H	−67.53	^b^	−29.41	^a^	−24.49	^b^	−51.25	^b^
WT	Diq_L	−46.99	^b^	−7.55	^a^	−15.65	^b^	−44.44	^b^
WT	Diq_H	−53.72	^b^	−21.69	^a^	−9.34	^ab^	−41.97	^b^

*Data are the % change of the mean. Different letters indicate significant differences between treatments with the control for each genotype at p < 0.05*.

### Tolerance of Transplastomic Lines Under *in vitro* NaCl Stress

**Figure 1:**
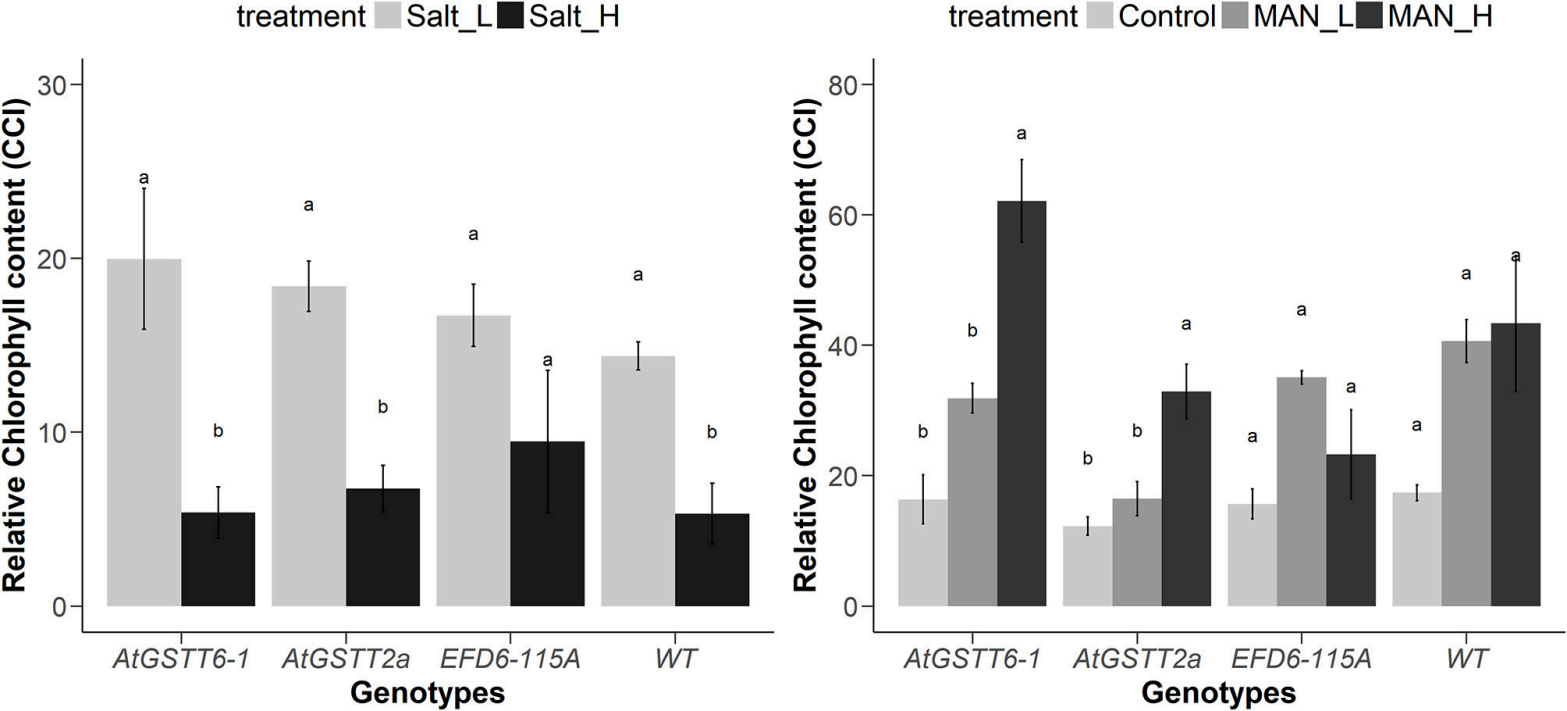
Changes in the relative chlorophyll content in pt^*At*GSTT^ (6-1 and 2a) and pt^EFD6−115A^ transplastomic lines, and WT plants growing under salinity (**left panel**) and drought (**right panel**) for 20 and 35 days, respectively. Different letters indicate significant differences between treatments for each genotype at *P* < 0.05 (*n* = 3 for control treatment and *n* = 6 for stress treatments).

Transplastomic pt^*At*GSTT^ line 6-1 and 2a, when grown in 150 mM NaCl, exhibited increased tolerance compared to WT plants (Figure S3, Table 3 and Table S4), with shoot length and M_F_ not showing statistically significant differences with the stress-free plants. Transplastomic line pt^*At*GSTT^6-1 also showed a non-statistically significant decrease in the shoot length even under the double salt concentration, 300 mM NaCl. Transplastomic line pt^EFD6−115A^ showed reduced M_F_ in both salinity concentrations compared to the stress-free plants, however showed statistically significant increase in root length at low NaCl concentration (Table S3 and Table S4). Root length and maximum quantum efficiency of PSII were only reduced under the 300 mM NaCl concentration in all transplastomic lines and WT plants (Table 3). Wild-type plants showed the lowest chlorophyll content in both low and high NaCl concentrations compared to the transplastomic lines, although not significantly different. Relative chlorophyll content was maintained in 300 mM NaCl concentration in the transplastomic line pt^EFD6−115A^, and it was reduced in all other genotypes including the WT compared to 150 mM NaCl concentration (Figure 1). Overall, the transplastomic line pt^*At*GSTT^2a demonstrated tolerance to both salt concentrations and especially at 150 mM NaCl as indicated by non-significant decrease in shoot length.

**Table 3:** Morphological parameters and maximum quantum yield of PSII (*F*v/*F*m) of GST transplastomic lines and WT tobacco plants grown for 20 days in salinity stress (150 and 300 mM NaCl) *in vitro*.

**Genotype**	**Treatment**	**Shoot length (cm)**	**_**HSD**_**	**Root length (cm)**	**_**HSD**_**	**M_**F (g)**_**	**_**HSD**_**	***F*v/*F*m**	**_**HSD**_**
pt^*At*GSTT^6-1	Control	2.13 ± 0.14	^a^	6.9 ± 0.45	^a^	2.23 ± 0.38	^a^	0.82 ± 0.004	^a^
pt^*At*GSTT^6-1	NaCl_L	1.68 ± 0.30	^a^	6.71 ± 0.67	^a^	1.47 ± 0.29	^ab^	0.83 ± 0.001	^a^
pt^*At*GSTT^6-1	NaCl_H	0.82 ± 0.10	^b^	2.41 ± 0.13	^b^	0.63 ± 0.1	^b^	0.78 ± 0.008	^b^
pt^*At*GSTT^2a	Control	3.23 ± 0.97	^a^	8.2 ± 0.97	^a^	2.6 ± 0.81	^a^	0.82 ± 0.003	^a^
pt^*At*GSTT^2a	NaCl_L	2.72 ± 0.44	^a^	6.9 ± 0.42	^a^	1.57 ± 0.22	^ab^	0.81 ± 0.002	^a^
pt^*At*GSTT^2a	NaCl_H	1.85 ± 0.28	^a^	2.26 ± 0.34	^b^	0.7 ± 0.12	^b^	0.78 ± 0.007	^b^
pt^EFD6−115A^	Control	3.4 ± 1.12	^a^	6.83 ± 0.59	^a^	2.51 ± 0.16	^a^	0.82 ± 0.012	^ab^
pt^EFD6−115A^	NaCl_L	1.5 ± 0.126	^b^	7.38 ± 0.62	^a^	1.6 ± 0.15	^b^	0.83 ± 0.001	^a^
pt^EFD6−115A^	NaCl_H	1.12 ± 0.19	^b^	2.78 ± 0.39	^b^	0.82 ± 0.21	^c^	0.78 ± 0.012	^b^
WT	Control	2.93 ± 0.29	^a^	7.26 ± 0.27	^a^	1.72 ± 0.12	^a^	0.82 ± 0.002	^a^
WT	NaCl_L	2.05 ± 0.22	^b^	6.78 ± 0.2	^a^	1.36 ± 0.1	^a^	0.83 ± 0.003	^a^
WT	NaCl_H	1.38 ± 0.15	^b^	1.86 ± 0.18	^b^	0.47 ± 0.05	^b^	0.76 ± 0.02	^b^

*Data (cm and g) are the mean ± SE (morphological data- control: n = 3 and treatments: n = 4; Fv/Fm: n = 6). Different letters indicate significant differences between treatments with the control for each genotype at p < 0.05*.

### Tolerance of Transplastomic Lines Under *in vitro* Mannitol Stress

**Figure 2:**
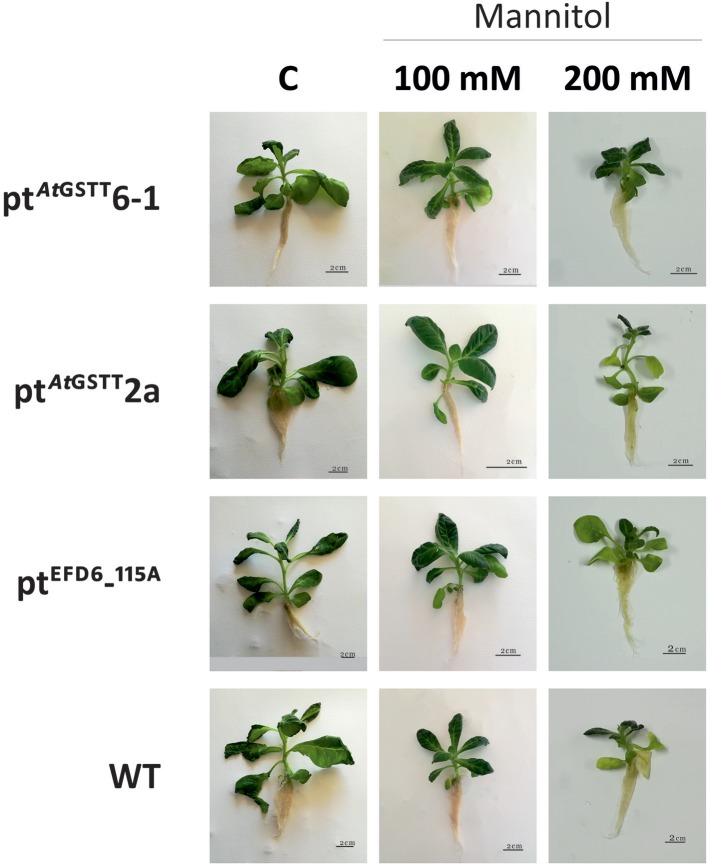
Effect of mannitol (100 and 200 mM) stress on growth of transplastomic lines and WT tobacco plants after 35 days in mannitol and stress-free (C) conditions.

Overexpression of the theta class *At*GSTT in chloroplasts increased PS II functionality in both mannitol concentrations (100 and 200 mM) compared to the stress-free plants and relative to pt^EFD6−115A^ and WT plants, which only increased the *F*v/*F*m at low mannitol stress and reduced their quantum yield under high osmotic stress (Table 4 and Table S5). Additionally, the pt^*At*GSTT^ lines showed increased chlorophyll content in 200 mM mannitol compared to pt^EFD6−115A^ and WT plants, which maintained their relative chlorophyll content in similar levels to the control plants (Figure 2). With respect to the effect of mannitol on growth, only pt^*At*GSTT^2a increased the shoot and root length in 200 mM mannitol, yet not significantly, in comparison to the stress-free plants, whilst the other transplastomic lines and the WT plants reduced the shoot length in high mannitol treatment. All transcriptomic lines reduced their M_F_ in the high mannitol stress, yet this reduction was less severe compared to the WT plants. Interestingly, in low mannitol stress only line pt^*At*GSTT^6.1 showed a non-significant reduction in the M_F_ and increased the shoot length and *F*v/*F*m, compared to the stress-free plants and the other transplastomic lines and WT plants (Table 4 and Table S5).

**Table 4:** Morphological traits and maximum quantum yield of PSII (*F*v/*F*m) of transplastomic lines and WT tobacco plants grown for 35 days in osmotic stress (100 and 200 mM mannitol stress).

**Genotype**	**Treatment**	**Shoot length (cm)**	**_**HSD**_**	**Root length (cm)**	**_**HSD**_**	**M_**F (g)**_**	**_**HSD**_**	***F*v/*F*m**	**_**HSD**_**
pt^*At*GSTT^6-1	Control	2.33 ± 0.03	^ab^	7.96 ± 0.56	^a^	2.7 ± 0.73	^a^	0.77 ± 0.01	^b^
pt^*At*GSTT^6-1	Man_L	2.73 ± 0.19	^a^	7.18 ± 0.36	^a^	2.15 ± 0.08	^a^	0.83 ± 0.001	^a^
pt^*At*GSTT^6-1	Man_H	2 ± 0.12	^b^	6.68 ± 0.31	^a^	0.96 ± 0.12	^b^	0.81 ± 0.005	^a^
pt^*At*GSTT^2a	Control	2.9 ± 0.36	^a^	7.2 ± 0.47	^a^	4.06 ± 0.57	^a^	0.79 ± 0.002	^a^
pt^*At*GSTT^2a	Man_L	0.52 ± 0.12	^b^	1.8 ± 0.55	^b^	0.56 ± 0.11	^b^	0.79 ± 0.005	^a^
pt^*At*GSTT^2a	Man_H	3.38 ± 0.46	^a^	8.85 ± 1.27	^a^	1.18 ± 0.14	^b^	0.75 ± 0.05	^a^
pt^EFD6−115A^	Control	6.1 ± 0.17	^a^	7.03 ± 0.37	^a^	4.63 ± 0.74	^a^	0.793 ± 0.001	^b^
pt^EFD6−115A^	Man_L	2.9 ± 0.29	^b^	6.63 ± 1.19	^a^	1.68 ± 0.11	^b^	0.823 ± 0.002	^a^
pt^EFD6−115A^	Man_H	2.23 ± 0.09	^b^	6.95 ± 0.39	^a^	1.11 ± 0.09	^b^	0.805 ± 0.008	^b^
WT	Control	5 ± 0.35	^a^	6.96 ± 0.43	^a^	5.11 ± 0.66	^a^	0.81 ± 0.002	^ab^
WT	Man_L	2.33 ± 0.18	^b^	7.13 ± 0.42	^a^	2.5 ± 0.22	^b^	0.82 ± 0.001	^a^
WT	Man_H	2.32 ± 0.1	^b^	7.51 ± 0.3	^a^	1.44 ± 0.13	^c^	0.79 ± 0.01	^b^

*Data are the mean ± SE (morphological data- control: n = 3 and treatments: n = 4; Fv/Fm: n = 6). Different letters indicate significant differences between treatments with the control for each genotype at p < 0.05*.

### Effect of GST Overexpression to the Transcriptome in Control and High Mannitol Stress

Based on the results the overexpression of the *At*GSTT in the transplastomic 2a line resulted in enhanced tolerance to both salinity concentrations and osmotic stress (200 mM mannitol), along with tolerance to herbicide induced oxidative stress based on the increase in M_D_ and relative chlorophyll content. Taking into consideration the osmotic component of salinity, we have selected this line for further investigation of the changes occurred in transcriptome and metabolome level under osmotic stress as it looked to be the most promising one for acquired stress tolerance to investigate the whole transcriptome and metabolome response of this line in order to understand in a systemic way the response of the transplastomic line.

**Figure 3:**
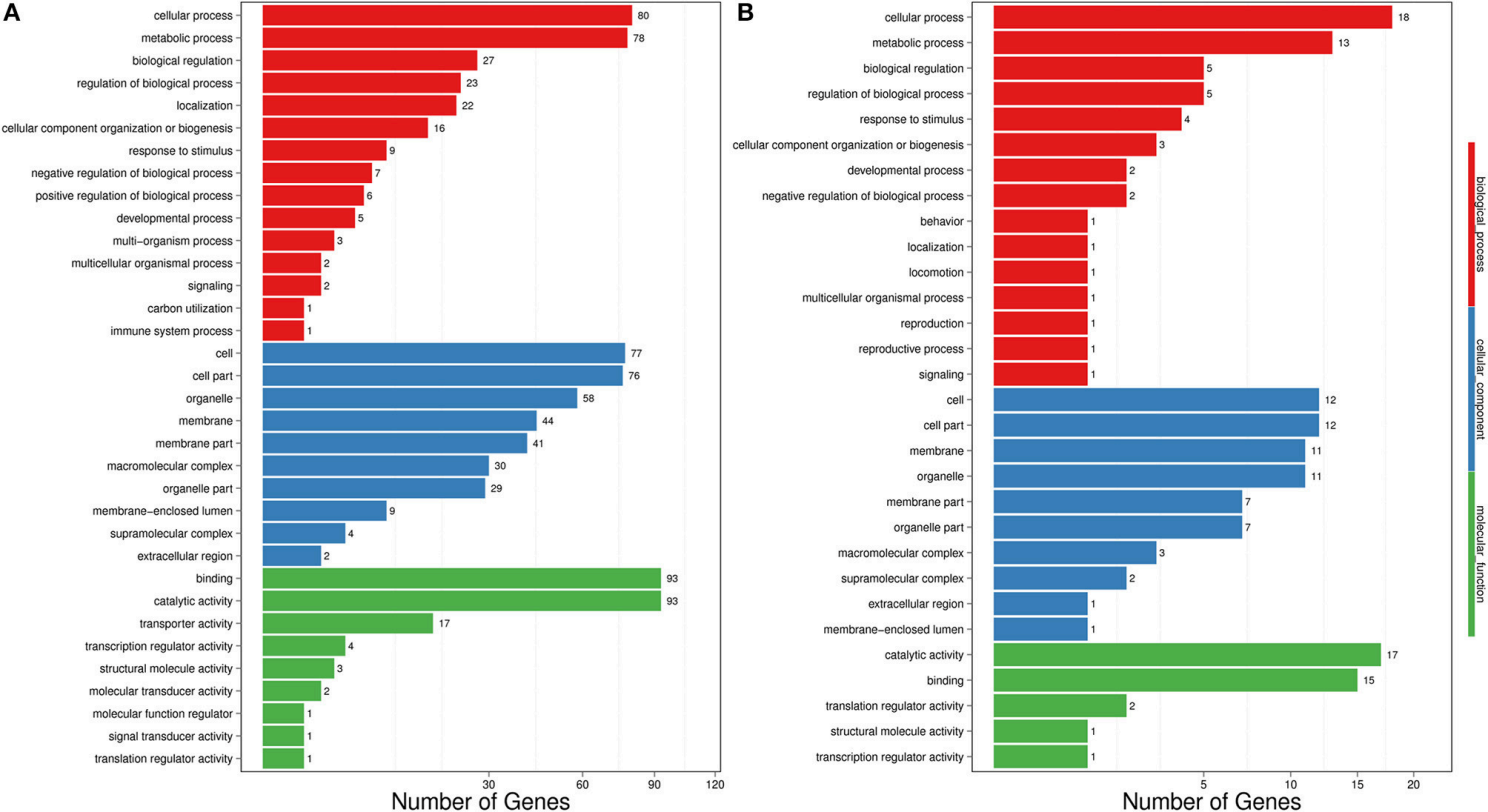
Histogram representation of Gene Ontology classification. **(A)** control conditions **(B)** under high mannitol stress (200 mM).

**Figure 4:**
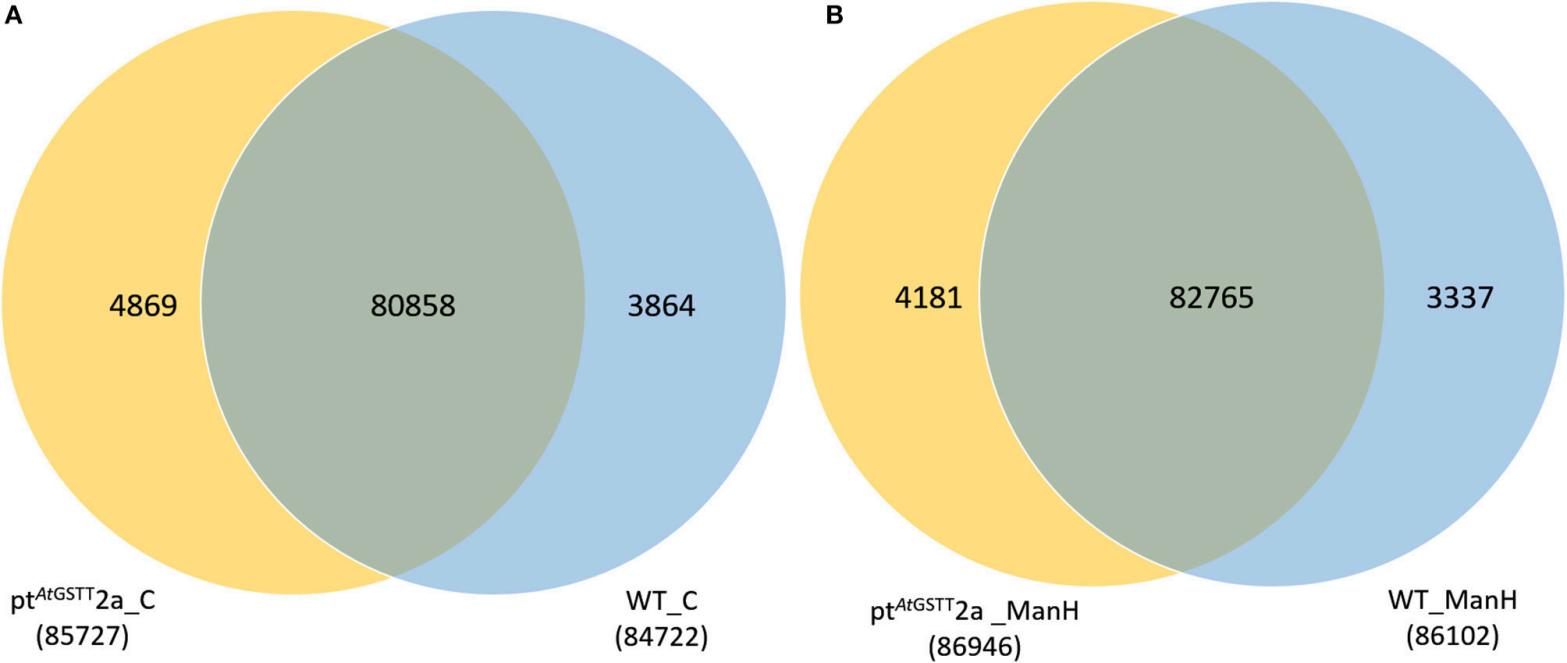
Venn diagram of differentially expressed genes. Comparison among **(A)** pt^*At*GSTT^2a and WT in control conditions (groups 1 and 3) and **(B)** pt^*At*GSTT^2a and WT in high mannitol (200 mM) stress (groups 2 and 4).

The transcriptome data were analyzed with RNA-Seq technology based on which, we performed the analysis of variance. The differential expression gene was selected according to the standard of *P* < 0.05 and the false discovery rate (FDR) was set to 0.001 to determine the threshold of the *P*-value for multiple tests. The absolute value of |log_2_Ratio| ≥ 1 was used to determine the difference between the gene expression transcription group and the database. Gene function, annotation, and classification were researched by GO analysis (Figures 3A,B). The RNA analysis through next generation sequencing of the entire transcriptome of the pt^*At*GSTT^2a and WT plants under control conditions and under high mannitol (osmotic stress) was studied (each sample in duplicate). The comparison between the two samples in each group showed that the expression profile was similar, thus allowing their combination and their analysis. The analysis generated 47.105 million clean reads in total with a Q20 (%) 98.38%. The reads generated a total of 80.623 transcripts of which 51.879 are known genes and 28.744 are unknown genes. On average 93.55% reads are mapped, and the uniformity of the mapping result for each sample suggests that the samples are comparable. Analysis of differentially expressed genes (DEGs) (Table S6) between the transplastomic plants under control conditions (group 1) and WT under control conditions (group 3) showed that there are 80858 commonly expressed DEGs while there 4869 unique DEGs expressed in pt^*At*GSTT^2a and 3864 in WT (Figure 4A). Furthermore, when we applied the mannitol (osmotic stress simulating drought) we found 82765 commonly expressed DEGs, 4181 in pt^*At*GSTT^2a plants, and 3337 in WT plants, thus there is a difference of 1907 more common DEGs, 688 fewer DEGs in pt^*At*GSTT^2a plants, and 527 fewer DEGs in WT plants under stress showing a reduction in differentially expressed genes both in WT and pt^*At*GSTT^2a plants (Figure 4B).

**Figure 5:**
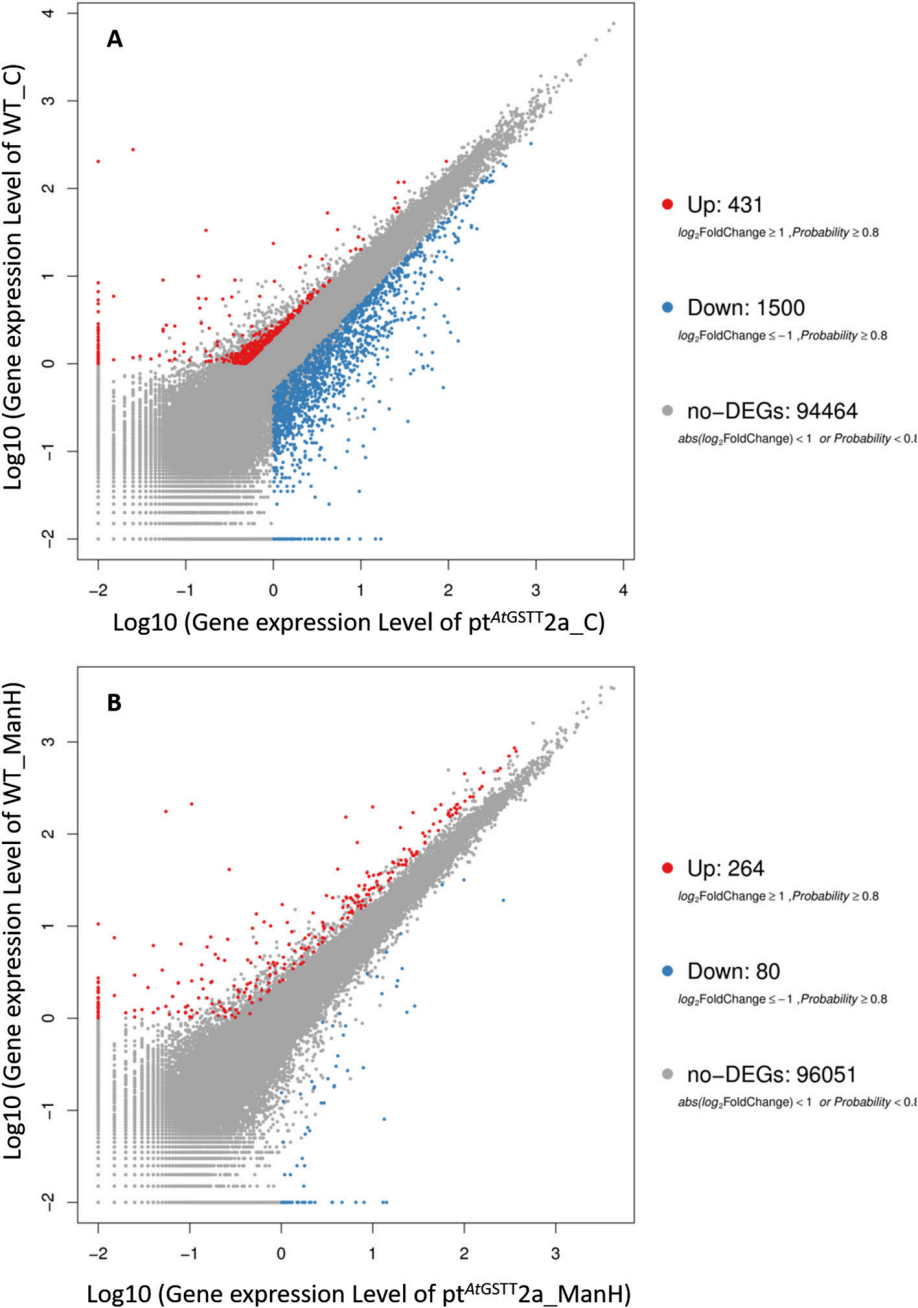
Scatter plot of differentially expressed genes in **(A)** pt^*At*GSTT^2a and WT in control conditions (groups 1 and 3), and **(B)** pt^*At*GSTT^2a and WT in high mannitol (200 mM) stress (groups 2 and 4).

Regarding the differentially expressed genes, between pt^*At*GSTT^2a and WT in control conditions (groups 1 and 3), we depicted 431 DEGs that were upregulated and 1500 downregulated (Figure 5A). Moreover, it is important to mention that between pt^*At*GSTT^2a and WT in high mannitol (200 mM) stress (groups 2 and 4), which are the samples under high mannitol stress only 264 were upregulated and 80 were downregulated (Figure 5B; Table S6).

Analysis of pt^*At*GSTT^2a overexpressing line and WT plants before the application of the high mannitol (osmotic stress) showed that genes like alanine transaminase and glutamate decarboxylase both implicated in alanine metabolism and biosynthesis were upregulated in pt^*At*GSTT^2a under control conditions. Additionally, 2,3-bisphosphoglycerate-dependent, phosphoglycerate mutase, glycine hydroxymethyl transferase were also upregulated whilst phosphoserine phosphatase was downregulated in the pathway of Glycine, serine, and threonine metabolism. Thus, high glycine content should be expected in pt^*At*GSTT^2a plants under control conditions.

Histone-lysine N-methyltransferase ASH1L, was down regulated in pt^*At*GSTT^2a plants whereas in histidine metabolism genes responsible for phosphoribosyl-ATP pyrophosphohydrolase, phosphoribosyl-AMP cyclohydrolase, histidinol dehydrogenase, and histidine decarboxylase were upregulated, suggesting that histidine should be accumulated in the pt^*At*GSTT^2a plants. Glutamate decarboxylase implicated in the Alanine, aspartate and glutamate metabolism and taurine and hypotaurine metabolism was upregulated in the transplastomic lines. Furthermore, glutathione S-transferases were found to be overexpressed (BGI_novel_G036737 K00799: BGI_novel_G036737 (-2.6), BGI_novel_G008526 (-2.3), BGI_novel_G023189 (-2.3).

An important metabolic pathway related to stress tolerance is starch and sucrose metabolism where trehalose 6-phosphate synthase was downregulated in pt^*At*GSTT^2a line under control conditions suggesting that plants were in a state of stress-priming. Transplastomic line pt^*At*GSTT^2a upregulated inositol polyphosphate 5-phosphatase and inositol-pentakis phosphate 2-kinase in the Inositol phosphate metabolism and Phosphatidylinositol signaling system. Alanine transaminase involved in Alanine, aspartate, and glutamate metabolism was upregulated, whilst glycine hydroxyl methyltransferase and phosphoserine phosphatase were found to be downregulated in the biosynthesis of amino acids pathway.

In the comparison of pt^*At*GSTT^2a vs. WT plants under the high mannitol stress the number of transcripts for pt^*At*GSTT^2a and WT plants was similar to those in control conditions; however, there were only 264 DEGs upregulated and 80 downregulated compared with 431 and 1500, respectively, in control conditions (Table S6). Important genes found with altered expression are glycerate dehydrogenase and hydroxypyruvate reductase upregulated in Glycine, serine and threonine metabolism as well as in DNA repair pathway, which is expected as stress produces ROS, to also affect nucleic acids. Additionally, the gene responsible for spermidine synthase implicated in glutathione metabolism, cysteine, and methionine metabolism and in arginine and proline metabolism was downregulated along with pectinesterase, an important gene implicated in Pentose and glucuronate interconversions as well as in cell wall degradation, in pt^*At*GSTT^2a compared to WT plants under stress. In the Phenylalanine, tyrosine and tryptophan biosynthesis pathway, the genes encoding bifunctional anthranilate synthase/indole-3-glycerol-phosphate synthase (G005943) related to tryptophane biosynthesis were upregulated as was the 5-methyltetrahydrofolate-homocysteine methyltransferase (G005943), leading to methionine.

### Effect of GST Overexpression in the Chloroplast to the Metabolome in High Mannitol Stress

**Figure 6:**
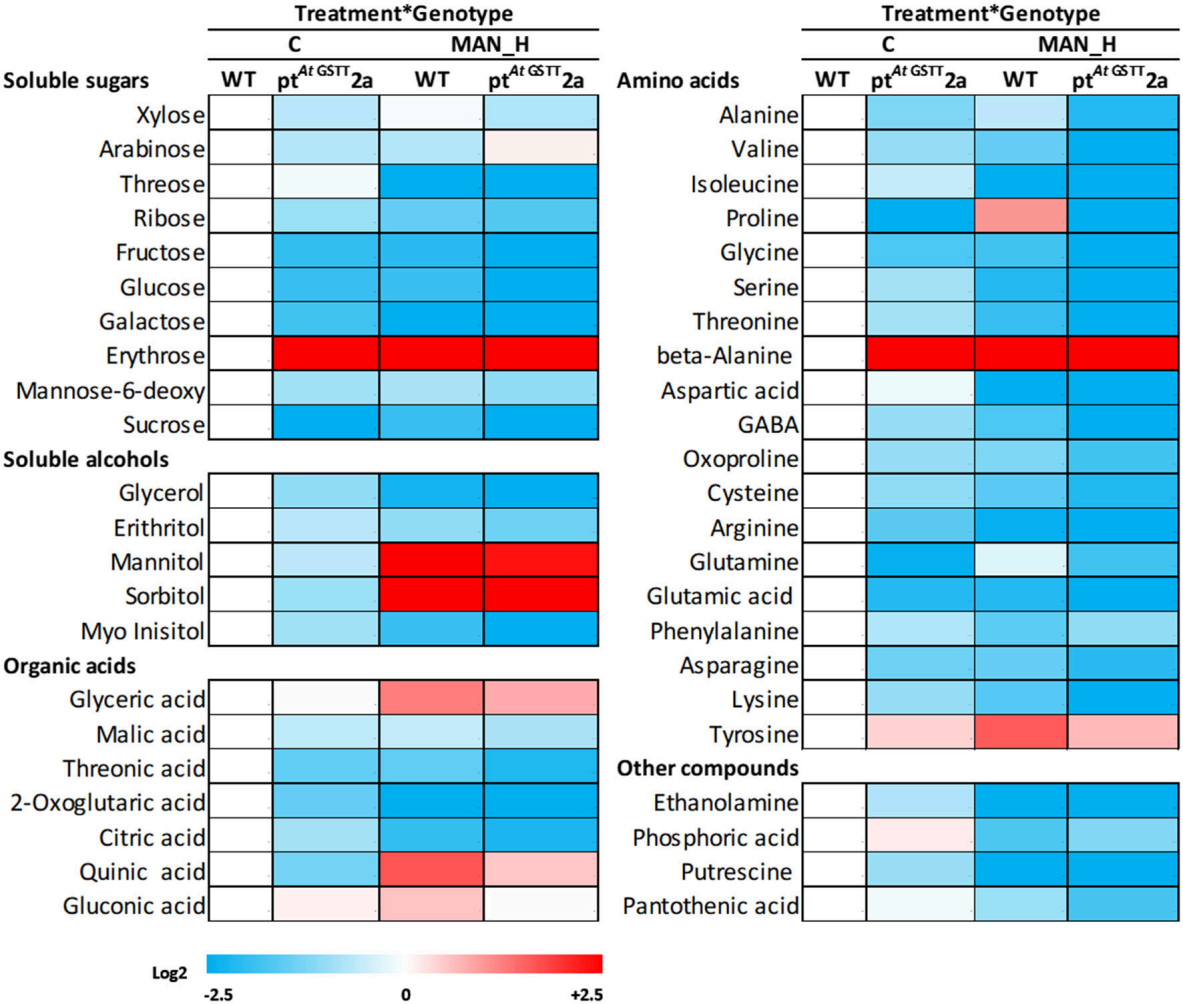
Heat map of primary metabolites of pt^*At*GSTT^2a and WT plants under high mannitol (200 mM) stress compared to WT control plants. Increase is indicated as red and decrease as blue (see color scale). Mean values of 3 independent determinations for each treatment were expressed as relative abundance compared to internal standard adonitol and are reported relative to the respective M_F_. Actual data are provided in Table S1.

The response of transplastomic line pt^*At*GSTT^2a and the WT plants was investigated further through the induced metabolic alterations. A total of 51 polar metabolites were identified (Figure 6; Table S1), of which 11 were soluble sugars, 5 soluble alcohols, 9 organic acids, 21 amino acids, and 5 other compounds (Figure 6; Tables S1, S2). The differences between the transplastomic line and WT plants under stress-free and high mannitol stress, revealed that ~78, 66.6, and 90% of the metabolic changes occurred due to treatment, genotypic, and treatment x genotype interaction effects, respectively.

In stress-free conditions, the overexpression of the *At*GSTT had a significant effect to the metabolic profile of transplastomic plants as indicated by the 27 out of 51 metabolites being significantly altered from the WT plants, 26 of which were down-regulated and only the benzoic acid was up-regulated (27.6-fold) (Table 5). The downregulated metabolites were mostly amino acids (13), such as proline, oxoproline, and valine, organic acids (5), such as citric, quinic, and threonic acids, soluble sugars (5), such as sucrose, fructose, and glucose and the soluble alcohols, erythritol, myo-inositol and glycerol. In contrast, the effect of high mannitol stress on plants overexpressing the GST chimera was moderate as only 16 metabolites were significantly changed. More specifically, plants overexpressing the GST chimera up-regulated only the soluble sugars threose (9.5-fold) and arabinose (0.82-fold), whilst, 14 metabolites were downregulated compared to the WT plants (Table 5). These results indicate that pt^*At*GSTT^2a transplastomic line was osmotolerant and able to maintain cellular homeostasis in comparison to the WT plants that required more energy to tolerate high mannitol stress.

**Table 5:** Metabolites that were significantly (*p* < 0.05) altered in pt^*At*GSTT^2a and WT plants under high mannitol (200 mM) and control conditions.

**pt**^*******At***GSTT****^**2a C/ WT C**		**pt**^*******At***GSTT****^**2a MAN_H/ WT MAN_H**	
**Metabolites**	**Fold-change**	**Metabolites**	**Fold-change**
Benzoic acid	27.6	Threose	9.5
Erithritol	−0.37	Arabinose	0.82
Xylose	−0.37	Serine	−0.31
Citric acid	−0.45	Putrescine	−0.46
Threonine	−0.45	Quinic acid	−0.54
Serine	−0.45	Glucose	−0.56
Myo-inositol	−0.46	Valine	−0.56
Putrescine	−0.49	Fructose	−0.57
Oxoproline	−0.5	Glycine	−0.65
Lysine	−0.5	Glutamine	−0.66
Valine	−0.51	Galactose	−0.68
Glycerol	−0.51	Sorbitol	−0.69
Cysteine	−0.53	Sucrose	−0.75
Alanine	−0.57	Proline	−0.94
Quinic acid	−0.61	Glycerol	−0.99
Asparagine	−0.62	Lysine	−1
Citruline	−0.64		
2-Isopropylmalic acid	−0.64		
2-Oxoglutaric acid	−0.64		
Threonic acid	−0.65		
Arginine	−0.67		
Glycine	−0.69		
Galactose	−0.72		
Glucose	−0.74		
Fructose	−0.75		
Sucrose	−0.82		
Proline	−0.85		

**Figure 7:**
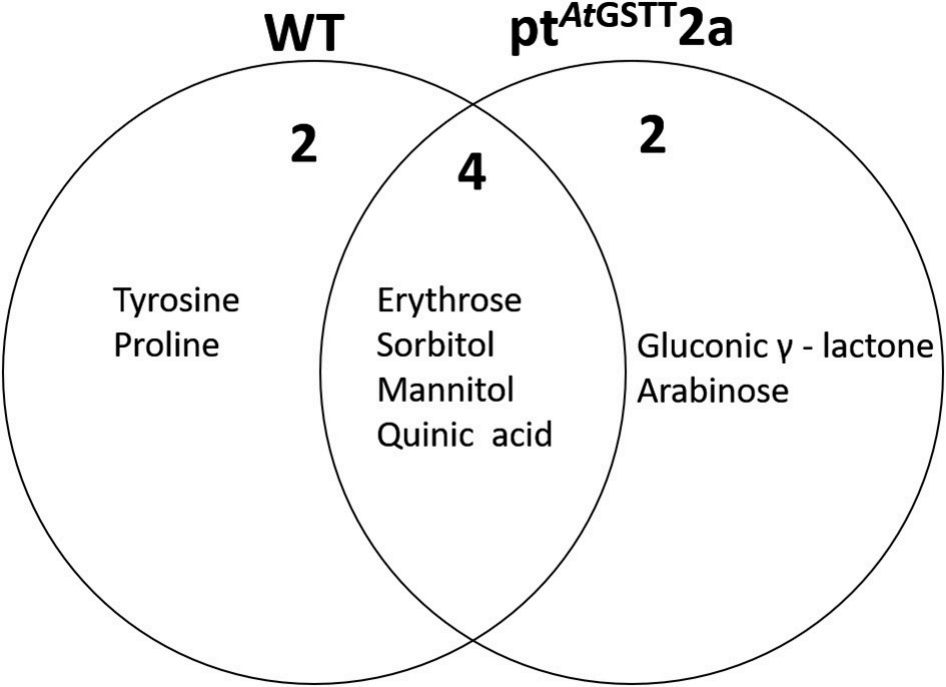
Venn diagram representation of metabolites commonly or differentially increased in the leaves of WT and pt^*At*GSTT^2a tobacco plants under high mannitol (200 mM) compared to non-stressed plants.

**Figure 8:**
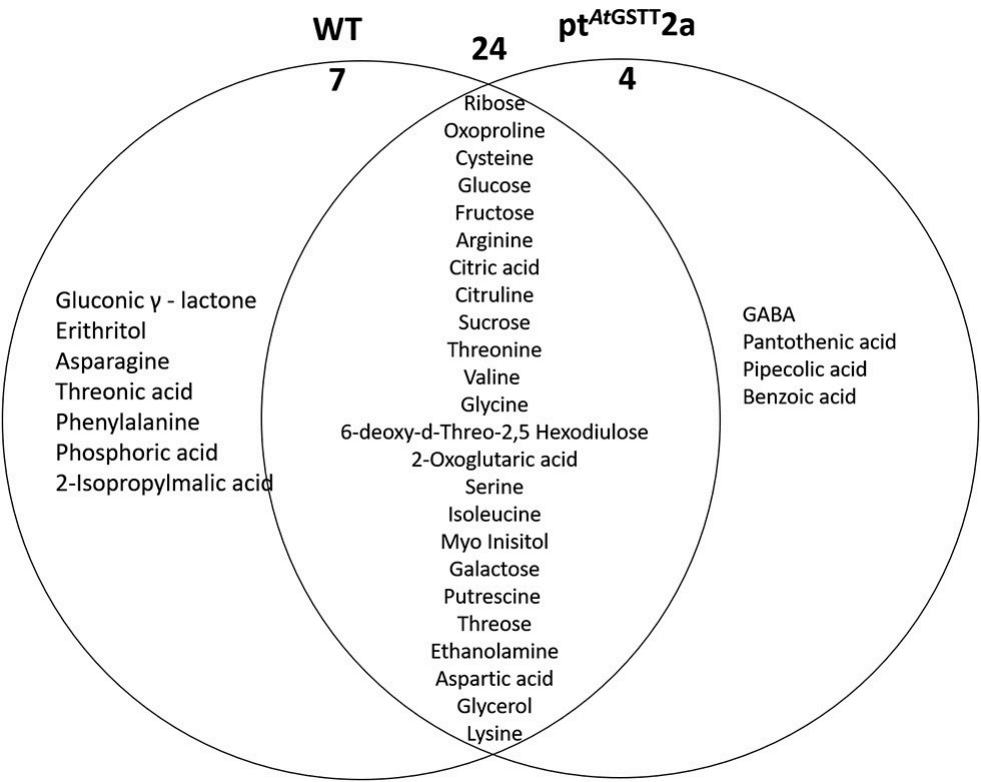
Venn diagram representation of metabolites commonly or differentially decreased in the leaves of WT and pt^*At*GSTT^2a tobacco plants under high mannitol (200 mM) compared to non-stressed plants.

The pt^*At*GSTT^2a line under high mannitol stress significantly altered more metabolites (38) compared to the 34 metabolites of the WT plants (Figures 7, 8; Table S7). The pt^*At*GSTT^2a line upregulated six metabolites of which four were common. In the increased metabolites two were soluble sugars, such as arabinose, which was unique for the transplastomic line, two were soluble alcohols, such as mannitol, and quinic acid (Figure 7). Erythrose and sorbitol were accumulated in greater concentrations in the WT plants than in the transplastomic line under high mannitol compared to control conditions (Table S7). Interestingly, the compatible solute mannitol was accumulated in greater concentration in the pt^*At*GSTT^2a by 6.84-fold compared to the 4.73-fold increase in the WT plants.

The WT plants down-regulated 31 metabolites compared to the 28 of the pt^*At*GSTT^2a plants. Among the metabolites that were decreased, 24 were common (6 soluble sugars, 12 amino acids, and 2 organic acids), such as the TCA cycle intermediate citric acid and the precursor of various amino acids aspartic acid (Figure 8; Table S7). Additionally, the WT plants had more differentially decreased metabolites compared to the pt^*At*GSTT^2a plants (Figure 8).

## Discussion

The adaptation response mechanisms of plants to adverse abiotic stresses result in the up-regulation of the reactive oxygen species (ROS) detoxification network, to mitigate the negative effects of oxidative stress, commonly induced under such conditions (Gill and Tuteja, 2010; Nianiou-Obeidat et al., 2017). Enhancing the ROS scavenging capacity in plants by direct gene expression in the chloroplast, an active cell compartment could in theory increase the photosynthetic rate and thus increase in yield, yet this is only a speculation that needs thorough investigation, which is beyond the scope of this research. The functional role of tobacco lines overexpressing the *At*GSTT in chloroplasts has been previously characterized (Dixon et al., 2008), yet, the roles of this *At*GSTT in plant homeostasis and response mechanisms both under abiotic and herbicide-induced oxidative stresses, and non-stress conditions are still required to be unraveled. Targeting the chloroplasts, we have assessed the osmotic, ionic, and oxidative potential of the pt^*At*GSTT^ lines and the *Zm*GSTU1-*Zm*GSTU2 chimera overexpressing line in comparison to WT plants. Our work shows that pt^*At*GSTT^ lines were tolerant to herbicide-induced oxidative and salinity stresses and showed enhanced response tolerance to mannitol-induced osmotic stress compared to WT plants.

The mode of bipyridiniums action is within the chloroplast by diverting electrons from photosystem I (PSI) of photosynthesis to form the Diquat radical, which in turn generates a highly destructive superoxide radical (Devine et al., 1992; Hawkes, 2014). Despite the observed reduction in maximum quantum yield of PSII, the transplastomic lines pt^*At*GSTT^ showed differential response mechanism in the accumulation of relative chlorophyll content in both half- and recommended field dose of Diquat, possibly to alleviate the negative effect of oxidative damage on PSII, which was apparent under all levels of Diquat in WT plants. It has been observed that transplastomic tobacco plants expressing simultaneously DHAR:GR and GST:GR showed enhanced tolerance to paraquat induced oxidative stresses while expression of either single transgene did not (Le Martret et al., 2011). Transplastomic overexpression of glutathione peroxidase (GP) in tobacco plants has shown to confer moderate tolerance to paraquat (Yoshimura et al., 2004), whereas, transplastomic tobacco lines overexpressing an *Escherichia coli* glutathione reductase (*gor*) gene have not enhanced protection from paraquat induced photooxidative stress (Poage et al., 2011). In the present study pt^*At*GSTT^ lines also had enhanced turgor maintenance in contrast to WT plants, which showed extensive dehydration, since Diquat is a rapid desiccant (Cronshey, 1961; Hawkes, 2014).

At 150 mM of NaCl concentration, both pt^*At*GSTT^ transplastomic lines showed enhanced growth (shoot length and M_F_) and pt^*At*GSTT^2a moderate tolerance to high salinity stress (300 mM NaCl) by maintaining the shoot length compared to WT plants. Additionally, under both NaCl concentrations, all transplastomic lines demonstrated a higher relative chlorophyll content compared to the WT plants. Similar results were observed in transplastomic tobacco plants overexpressing a choline monooxygenase (*Bv*CMO) from beetroot which increased photosynthetic rate and apparent quantum yield of photosynthesis in the presence of 150 mM NaCl when compared to WT, and the maximal efficiency of PSII photochemistry in both wild type and transplastomic plants was not affected (Zhang et al., 2008). This is also consistent with our results indicating that the overexpression of the *At*GSTT and the *ZmGSTU1-ZmGSTU2* chimera can protect PSII reaction centers from damage. Transplastomic carrot plants expressing the *badh* gene demonstrated enhanced tolerance up to up to 400 mM NaCl compared to untransformed plants exhibiting severe growth inhibition at 200 mM NaCl (Kumar et al., 2004). Herein, the pt^*At*GSTT^ transplastomic lines pt^*At*GSTT^2a and especially pt^*At*GSTT^6-1 demonstrated enhanced photo-tolerance when exposed to 200 mM mannitol stress demonstrating increased relative chlorophyll content and maximum yield of PSII compared to WT plants. Increasing or maintaining the chlorophyll content in transgenic chloroplasts suggests the integrity of thylakoid membranes, even in the presence of high concentrations of NaCl and mannitol, demonstrating the advantage of overexpressing the *At*GSTT in the chloroplasts. Similar results were observed by Lee et al. (2003).

The transcriptomics analysis of pt^*At*GSTT^2a line and WT plants under control and high mannitol stress suggests that plants of the pt^*At*GSTT^2a overexpressing line, before the application of the high mannitol (osmotic stress) upregulated genes related to stress tolerance such as genes encoding for alanine transaminase and glutamate decarboxylase both implicated in alanine metabolism and biosynthesis. Alanine was found to be the main amino acid accumulated in M*edicago truncatula* seedlings under hypoxic stress (Limami et al., 2008). Furthermore, genes encoding for 2,3-bisphosphoglycerate-dependent phosphoglycerate mutase and glycine hydroxymethyl transferase were also upregulated, while phosphoserine phosphatase was downregulated in glycine, serine and threonine metabolism, which may lead to increased glycine. Therefore, high glycine content should be expected in pt^*At*GSTT^2a plants under control conditions but considering that high glycine content is correlated with stress tolerance and especially drought resistance (Thankur and Rai, 1982), the pt^*At*GSTT^2a line is probably in a stress primed state before the application of the stress. . Also, glutamate decarboxylase is upregulated which is implicated in Alanine, aspartate and glutamate metabolism and in taurine and hypotaurine metabolism and was also confirmed in metabolomics analysis. Interestingly, in the resurrection plant *Sporobolus stapfianus* Martinelli et al. (2007) reported that the accumulation of asparagine and glutamate might have led to its conversion to arginine and asparagine, as all of the above are considered to play important role in plant protection against drought stress (Martinelli et al., 2007). Moreover, we found that the histone-lysine N-methyltransferase ASH1L, was down regulated in pt^*At*GSTT^2a plants in histidine metabolism whereas, genes responsible for histidine metabolism like phosphoribosyl-ATP pyrophosphohydrolase phosphoribosyl-AMP cyclohydrolase, histidinol dehydrogenase and histidine decarboxylase were upregulated, suggesting that histidine should be accumulated in the pt^*At*GSTT^2a plants prior to the application of stress, which reinforces the notion that the transplastomic plants are in a primed condition as before (Tran et al., 2007; Witt et al., 2012). Similarly, trehalose 6-phosphate synthase was found to be downregulated in pt^*At*GSTT^2a line under control conditions highlighting the stress primed condition of the transplastomic line (Lee et al., 2003; Ilhan et al., 2015).

When the transplastomic line pt^*At*GSTT^2a and WT plants were exposed to osmotic stress (high mannitol), the number of transcripts for pt^*At*GSTT^2a and WT plants did not change compared to those in control conditions. However, important genes found with an altered expression such as those encoding for glycerate dehydrogenase and hydroxypyruvate reductase which were found to be upregulated in glycine, serine and threonine metabolism as well as in DNA repair pathway, which, as stress produces ROS is expected to affect nucleic acids. In contrast, the gene responsible for spermidine synthase implicated in glutathione metabolism, cysteine and methionine metabolism and in arginine, and proline metabolism was down regulated. Proline is important in stress tolerance and has been found to increase during different environmental stresses like salinity, drought, UV, and extreme temperatures (Ashraf and Foolad, 2007). In addition, polyamines like spermidine have been reported to play a role in inducing stress response under various stresses that produce ROS as they might serve as ROS scavengers, and as positive regulators for expression of stress response genes. Thus, polyamines like spermidine could perform as primal stress molecules in plants (Rhee et al., 2007). Additionally, pectinesterase was found to be downregulated in pt^*At*GSTT^2a compared to WT plants under stress. This is an important gene implicated in pentose and glucuronate interconversions as well as in cell wall degradation as it was found to be upregulated in plants exposed to permissive high temperature conditions (37°C). This parallels to acclimation in order to acquire thermotolerance as a result of the cell wall modification (Yang et al., 2006). However, the downregulation of such enzymes in the pt^*At*GSTT^2a line under mannitol stress suggests that these plants might be in a state of acclimation prior to the application of the stress. In the Phenylalanine, tyrosine and tryptophan biosynthesis pathway the genes encoding bifunctional anthranilate synthase/indole-3-glycerol-phosphate synthase (G005943) related to tryptophane biosynthesis were upregulated as was the 5-methyltetrahydrofolate-homocysteine methyltransferase (G005943), leading to methionine. In a rat model, actin oxidative damage by ROS was found to occur through the oxidation of cysteine, tryptophan and methionine (Fedorova et al., 2010). If this is also the case in plants, then increased amounts of these amino acids might be needed and thus, leading to the upregulation of the genes responsible for their production, as it was found herein; however, this hypothesis needs further investigation.

The metabolomics analysis was performed on transplastomic and WT plants grown under high mannitol stress and controlled conditions *in vitro* for 35 days. The overexpression of the *At*GSTT had a significant effect to the metabolic profile of transplastomic plants, since many metabolites were downregulated under both control and drought conditions indicating limited perturbation of metabolic homeostasis in the transplastomic lines. Especially under high mannitol stress the pt^*At*GSTT^2a line had higher concentrations of the soluble sugars, threose and arabinose, which demonstrates the protective role against osmotic stress (Keunen et al., 2013). The soluble alcohol mannitol was accumulated in greater concentration in pt^*At*GSTT^2a line despite the common increase in WT plants under high mannitol stress. In contrast to our results mannitol accumulation was decreased in transgenic tobacco plants overexpressing a *Gmgstu4* gene under salinity stress (Kissoudis et al., 2015b). Mannitol accumulation plays an important role in osmotic adjustment and signaling molecule enhance tolerance to water stress in various plant species (Slama et al., 2015). Additionally, the greater M_F_ and shoot length of pt^*At*GSTT^2a compared to the WT plants under mannitol stress indicates a possible relation between increase in mannitol and improved growth. Similar results were observed in peanut (Bhauso et al., 2014) and *Zea mays* (Nguyen et al., 2013) plants overexpressing *mtlD* genes, which conferred water-deficit stress tolerance by inducing the accumulation of mannitol and increase in biomass and relative water content under drought conditions.

The results above suggest that overexpression of the *At*GSTT in the chloroplasts resulted in enhanced photo-tolerance and turgor maintenance under herbicide-induced oxidative (increased M_D_ and Relative chlorophyll content) and salinity stresses (higher chlorophyll, non-significant decrease in shoot length and M_F_ compared to the control plants) and enhanced response tolerance to high mannitol-induced osmotic stress (increased shoot and root length). Whole-genome transcriptome analysis revealed that genes related to stress tolerance, such as GSTs, were upregulated in pt^*At*GSTT^2a line under both control and high mannitol stress conditions indicating an acclimation state to stress. In parallel, the metabolic profile indicated limited perturbations of the metabolic homeostasis in the transplastomic lines and greater accumulation of mannitol and soluble sugars under high mannitol stress. We have therefore established that the transplastomic plants overexpressing the pt^*At*GSTT^2a in the chloroplast are probably in a state of acclimation to stress, thus, when the actual stress is applied there is limited need for overexpression of the whole array of stress tolerance mechanisms, which is imprinted in the levels of relative gene expression. As mentioned before, we found only limited genes to be upregulated in the pt^*At*GSTT^2a transplastomic line compared to WT under stress conditions while at the same time we have found genes related to stress tolerance upregulated in pt^*At*GSTT^2a plants compared to WT in stress-free conditions, strengthening the hypothesis that the *AtGSTT* overexpressed in plastids might have conferred plant stress tolerance.

Challenges caused by climate change will demand for quick action of the scientific community in order to develop stress tolerant varieties to secure enough food for the increasing world population. GSTs, for have proven to be enzymes involved in stress tolerance (Dixon et al., 1998, 2008, 2011; Axarli et al., 2009, 2017; Chronopoulou and Labrou, 2009; Benekos et al., 2010; Chronopoulou et al., 2011, 2012, 2014; Madesis et al., 2013; Kissoudis et al., 2015a,b; Labrou et al., 2015; Lo Cicero et al., 2015, 2017; Nianiou-Obeidat et al., 2017) might help toward the development of plant acclimation to environmental stresses. In some cases, the overexpression of a single antioxidant enzyme might not provide protection against oxidative stress whilst, simultaneous expression of multiple antioxidant enzymes is more effective than a single expression for enhancing tolerance to environmental stresses (Le Martret et al., 2011). Herein, the *Zm*GSTU1-*Zm*GSTU2 chimera was able to induce photoprotection of the photosystem II under sever salinity stress, yet it was not as tolerant as the single AtGSTTs overexpressed in the chloroplasts. Potentially, the expression of multiple defense genes encoding enzymes belonging to different classes could generate plants with enhanced stress tolerance (Zhao and Zhang, 2006) able to withstand multiple stresses, which needs to be further investigated. This study provides evidence that overexpression of both the theta class *At*GSTT and the unique chimera GSTU1-GSTU2 from *Zea mays* in the chloroplast resulted in enhanced tolerance of the transplastomic plants to abiotic stresses. Furthermore, transcriptomics and metabolomics analysis showed that the GST overexpressing plants were in a stress tolerance priming state even before the application of the severe osmotic stress (high mannitol concentration) thus, enhancing the plant's ability to tolerate abiotic stresses.

## Author Contributions

ES designed and performed part of the experiments, and the statistical analysis, and wrote the manuscript. SG and AI performed part of the experiments. SK performed part of the experiments. MM performed part of the metabolomics and wrote part of the metabolomics section. EC performed part of the experiments and read the manuscript. RE wrote and edited part of the manuscript. AD wrote and edited the manuscript. NL, IN-O and PM designed the research, wrote and edited the manuscript.

### Conflict of Interest Statement

The authors declare that the research was conducted in the absence of any commercial or financial relationships that could be construed as a potential conflict of interest.
