# Supplementary material for: Tolerance of Transplastomic Tobacco Plants Overexpressing a Theta Class Glutathione Transferase to Abiotic and Oxidative Stresses
Source: Front Plant Sci. 2019 Jan 11;9:1861. doi: 10.3389/fpls.2018.01861 (PMC6337918; doi:10.3389/fpls.2018.01861)
Supplement: Supplementary file 1 [file Data_Sheet_1.docx]

Tolerance of transplastomic tobacco plants overexpressing a theta class glutathione transferase to abiotic and oxidative stresses

Stavridou E.^1^, Michailidis M.^2^, Gedeon S.^3^, Ioakeim A.^3^, Κostas S.^4^, Chronopoulou E.^5^, Labrou E. N. ^5^, Edwards R. ^6^ Day A.^7^, Nianiou-Obeidat I.^8^, Madesis P.^1*^


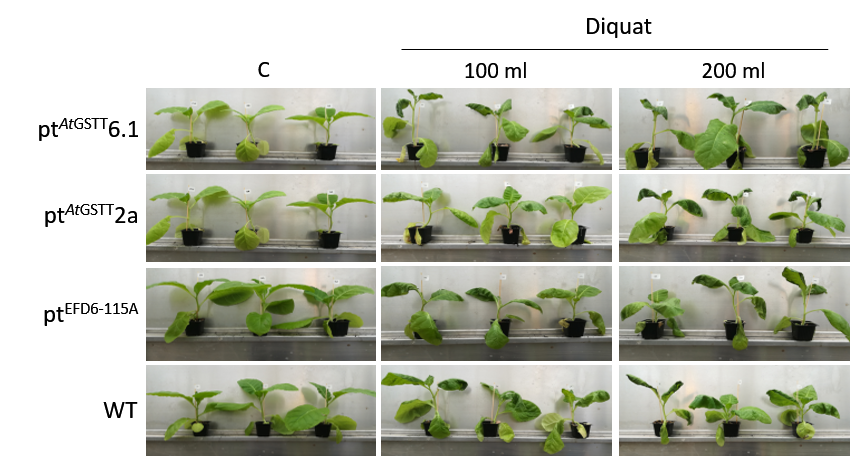


**Figure S1.** Effect of Diquat (100 and 200 ml per 30 L^-1^ 1000 m^-2^) on growth of transplastomic lines and WT tobacco plants after 1-day exposure to herbicide-induced oxidative stress and control (C) conditions.


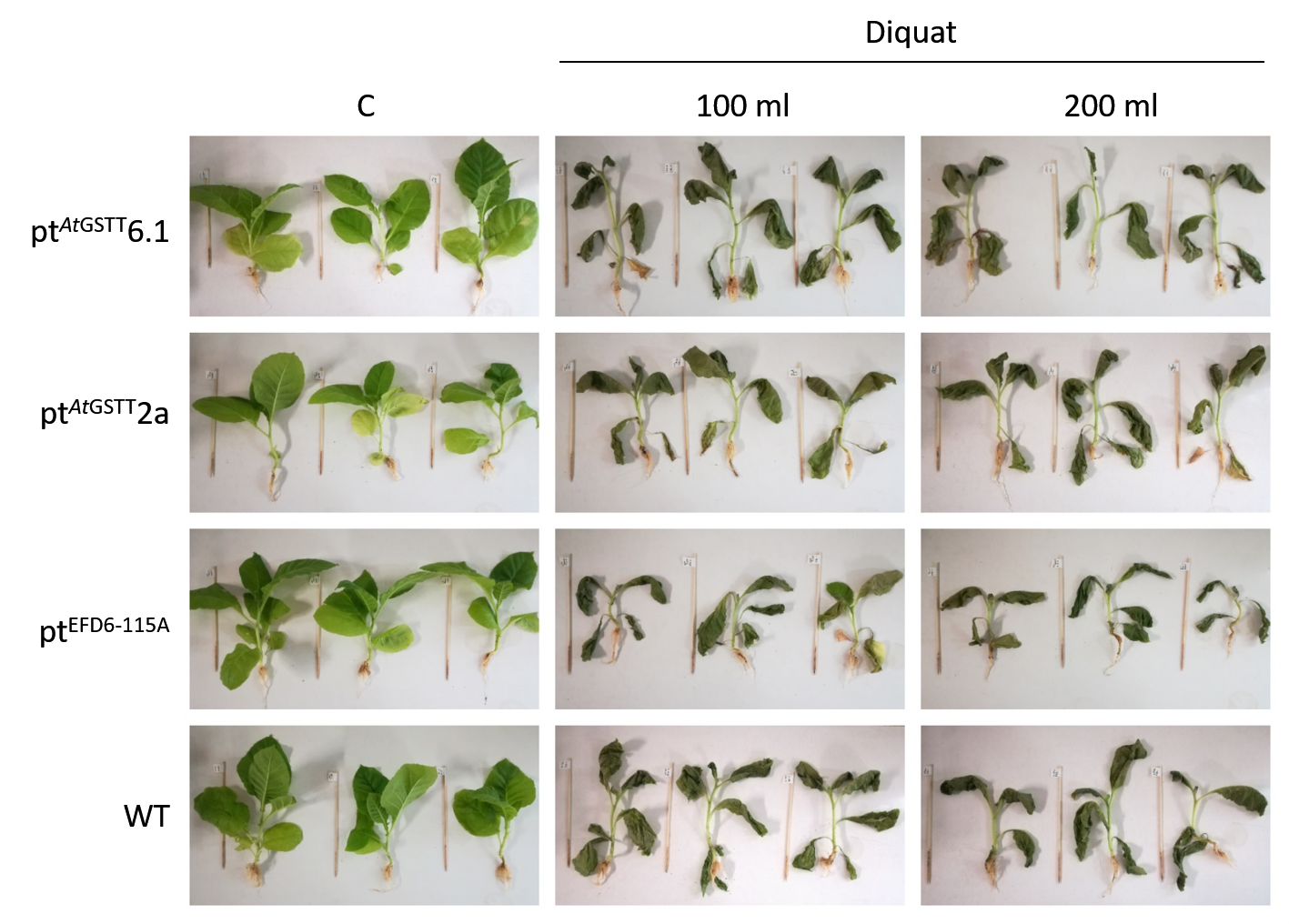


**Figure S2.** Effect of Diquat (100 and 200 ml per 30 L^-1^ 1000 m^-2^) on growth of transplastomic lines and WT tobacco plants after 2-days exposure to herbicide-induced oxidative stress and control (C) conditions.


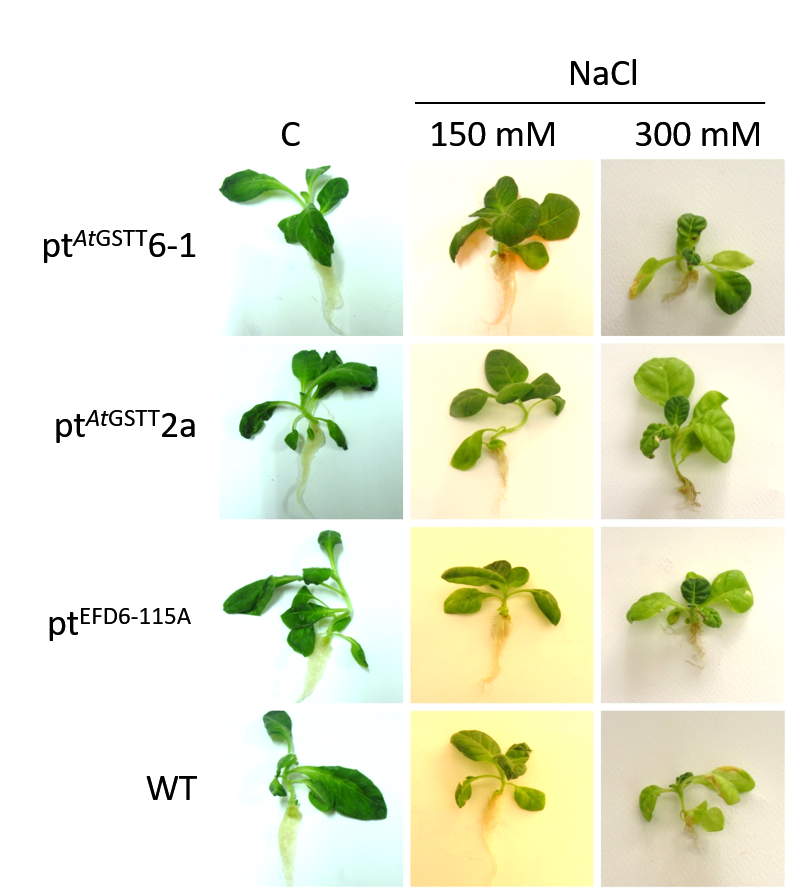


**Figure S3.** Effect of NaCl (150 and 300 mM) on growth of transplastomic lines and WT tobacco plants after 20 days under NaCl and control (C) conditions *in vitro*.

**Table S3.** Growth (fresh-M_F_ and dry-M_D_ matter; g) and photophysiological parameters (relative chlorophyll content- Chl and Maximum quantum yield of PSII-*F*v/*F*m) of *GST transplastomic* lines and WT tobacco plants grown for 2 days in low (Diq_L) and high (Diq_H) Diquat dose and control conditions (C). Data are mean ± SE (n=6) at p<0.05.

| **Genotype** | **Treatment** | **M_F (g)_** | **_HSD_** | **M_D (g)_** | **_HSD_** | **Chl** | **_HSD_** | ***F*v*/F*m** | **_HSD_** |
| --- | --- | --- | --- | --- | --- | --- | --- | --- | --- |
| pt*^At^*^GSTT^6.1 | Control | 10.7±2.11 | ^a^ | 0.81±0.15 | ^a^ | 6±0.203 | ^b^ | 0.8±0.003 | ^a^ |
| pt*^At^*^GSTT^6.1 | Diq_L | 5.9±0.99 | ^a^ | 0.75±0.15 | ^a^ | 7.48±0.386 | ^a^ | 0.47±0.078 | ^b^ |
| pt*^At^*^GSTT^6.1 | Diq_H | 5.2±0.29 | ^a^ | 0.62±0.064 | ^a^ | 8.11±0.523 | ^a^ | 0.44±0.079 | ^b^ |
| pt*^At^*^GSTT^2a | Control | 9.53±0.72 | ^a^ | 0.59±0.063 | ^a^ | 7.04±0.24 | ^a^ | 0.79±0.005 | ^a^ |
| pt*^At^*^GSTT^2a | Diq_L | 4.78±0.6 | ^b^ | 0.73±0.12 | ^a^ | 7.02±0.323 | ^a^ | 0.45±0.069 | ^b^ |
| pt*^At^*^GSTT^2a | Diq_H | 6.66±0.29 | ^a^ | 0.96±0.08 | ^a^ | 7.64±0.258 | ^a^ | 0.4±0.084 | ^b^ |
| pt^EFD6-115A^ | Control | 14.26±1.17 | ^a^ | 0.85±0.095 | ^a^ | 7.47±0.458 | ^a^ | 0.8±0.003 | ^a^ |
| pt^EFD6-115A^ | Diq_L | 4±0.096 | ^b^ | 0.61±0.037 | ^a^ | 6.06±0.256 | ^ab^ | 0.41±0.079 | ^b^ |
| pt^EFD6-115A^ | Diq_H | 4.63±0.81 | ^b^ | 0.6±0.077 | ^a^ | 5.64±0.329 | ^b^ | 0.39±0.085 | ^b^ |
| WT | Control | 13.96±1.84 | ^a^ | 1.06±0.13 | ^a^ | 8.56±0.286 | ^a^ | 0.81±0.0035 | ^a^ |
| WT | Diq_L | 7.4±0.78 | ^b^ | 0.98±0.14 | ^a^ | 7.22±0.439 | ^b^ | 0.45±0.08 | ^b^ |
| WT | Diq_H | 6.46±0.36 | ^b^ | 0.83±0.096 | ^a^ | 7.76±0.473 | ^ab^ | 0.47±0.082 | ^b^ |

**Table S4.** Percent of change difference in morphological traits (shoot and root length; cm and M_F_; g) and maximum quantum yield of PSII (*F*v/*F*m) of GST transplastomic lines and WT tobacco plants grown for 20 days in salinity stress (150 and 300 mM NaCl) *in vitro*. Data are the mean ± SE. Different letters indicate significant differences between treatments for each genotype at p<0.05.

| **Genotype** | **Treatment** | **shoot length_%_** | **_HSD_** | **root length_%_** | **_HSD_** | **M_F%_** | **_HSD_** | ***F*v/*F*m_%_** | **_HSD_** |
| --- | --- | --- | --- | --- | --- | --- | --- | --- | --- |
| pt*^At^*^GSTT^6.1 | NaCl_L | -21.13 | ^a^ | -2.75 | ^a^ | -34.08 | ^ab^ | 1.22 | ^a^ |
| pt*^At^*^GSTT^6.1 | NaCl_H | -61.50 | ^b^ | -65.07 | ^b^ | -71.75 | ^b^ | -4.88 | ^b^ |
| pt*^At^*^GSTT^2a | NaCl_L | -15.79 | ^a^ | -15.85 | ^a^ | -39.61 | ^ab^ | -1.22 | ^a^ |
| pt*^At^*^GSTT^2a | NaCl_H | -42.72 | ^a^ | -72.44 | ^b^ | -73.07 | ^b^ | -4.87 | ^b^ |
| pt^EFD6-115A^ | NaCl_L | -55.88 | ^b^ | 8.05 | ^a^ | -36.25 | ^b^ | 1.22 | ^a^ |
| pt^EFD6-115A^ | NaCl_H | -67.06 | ^b^ | -59.29 | ^b^ | -67.33 | ^c^ | -4.87 | ^b^ |
| WT | NaCl_L | -30.03 | ^b^ | -6.61 | ^a^ | -20.93 | ^a^ | 1.22 | ^a^ |
| WT | NaCl_H | -52.90 | ^b^ | -74.38 | ^b^ | -72.67 | ^b^ | -7.32 | ^b^ |

**Table S5.** Percent of change difference in morphological traits (shoot and root length; cm and M_F_; g) and maximum quantum yield of PSII (*F*v/*F*m) of GST transplastomic lines and WT tobacco plants grown for 35 days in osmotic stress (100 and 200 mM mannitol stress) *in vitro*. Data are the % change of the mean ± SE. Different letters indicate significant differences between treatments with the control for each genotype at p<0.05.

| **Genotype** | **Treatment** | **shoot length_%_** | **_HSD_** | **root length_%_** | **_HSD_** | **M_F%_** | **_HSD_** | ***F*v/*F*m_%_** | **_HSD_** |
| --- | --- | --- | --- | --- | --- | --- | --- | --- | --- |
| pt*^At^*^GSTT^6.1 | Man_L | 17.17 | ^a^ | -9.79 | ^a^ | -20.37 | ^a^ | 7.79 | ^a^ |
| pt*^At^*^GSTT^6.1 | Man_H | -14.16 | ^b^ | -16.08 | ^a^ | -64.44 | ^b^ | 5.19 | ^a^ |
| pt*^At^*^GSTT^2a | Man_L | -82.76 | ^b^ | -75 | ^b^ | -86.21 | ^b^ | 0 | ^a^ |
| pt*^At^*^GSTT^2a | Man_H | 16.55 | ^a^ | 22.92 | ^a^ | -70.93 | ^b^ | -5.06 | ^a^ |
| pt^EFD6-115A^ | Man_L | -52.46 | ^b^ | -5.68 | ^a^ | -63.71 | ^b^ | 3.79 | ^a^ |
| pt^EFD6-115A^ | Man_H | -63.44 | ^b^ | -1.14 | ^a^ | -76.03 | ^b^ | 1.26 | ^b^ |
| WT | Man_L | -53.4 | ^b^ | 2.44 | ^a^ | -51.07 | ^b^ | 1.23 | ^a^ |
| WT | Man_H | -53.6 | ^b^ | 7.9 | ^a^ | -71.82 | ^c^ | -2.47 | ^b^ |
